# A study on the morbidity patterns and healthcare utilization of tea garden workers of Siliguri, West Bengal, India

**DOI:** 10.1186/s12889-026-28744-9

**Published:** 2026-07-31

**Authors:** Suteerna Choudhury, Kumar Sumit

**Affiliations:** Department of Global Public Health Policy and Governance, Prasanna School of Public Health, Manipal Academy of Higher Education, Manipal, Karnataka 576104 India

**Keywords:** Tea plantation workers, Healthcare utilization, Morbidity burden, Occupational health, India

## Abstract

**Background:**

Tea garden workers in India, particularly in the Siliguri region of West Bengal, experience a high burden of morbidity and significant barriers to healthcare utilization due to hazardous occupational conditions, socio-economic vulnerabilities, and inadequate access to quality health services. This study aimed to assess morbidity patterns, examine healthcare utilization, and identify key determinants influencing health-seeking behaviour among tea garden workers.

**Methods:**

A cross-sectional study was conducted between January and March 2024, among 167 tea garden workers in Siliguri, West Bengal. Data were collected through interviewer-administered semi-structured questionnaires using a multistage random sampling approach. Descriptive statistics were used to summarize morbidity patterns and healthcare access. Chi-square tests examined associations between variables, while multivariable Poisson and binary logistic regression analyses were used to identify factors associated with morbidity burden and healthcare utilization. Ethical considerations, including informed consent and confidentiality, were ensured prior to data collection.

**Results:**

The study revealed a high prevalence of infectious, chronic, and occupational health conditions among tea garden workers. Self-reported tuberculosis (50.9%), diabetes mellitus (36.5%), and occupational injuries (67.7%) were among the most commonly reported conditions. Healthcare utilization remained suboptimal, with 29.5% of symptomatic workers not seeking formal healthcare and only 20.4% reporting utilization of government health schemes. Tea garden hospitals were the most frequently utilized healthcare facilities (57.4%).

**Conclusions:**

The findings highlight substantial health and healthcare challenges faced by tea garden workers in West Bengal. Targeted interventions are needed to improve healthcare accessibility, morbidity management, and occupational health conditions. Strengthening tea garden hospital infrastructure, enhancing workplace health programmes, improving enforcement of labour laws, and increasing awareness of government health schemes could help address existing gaps. Community-based health initiatives and financial support mechanisms may further contribute to improving the overall well-being of this marginalized workforce.

**Supplementary Information:**

The online version contains supplementary material available at https://doi.org/10.1186/s12889-026-28744-9.

## Introduction

The tea industry in India, spanning nearly 200 years, holds an important place in the national economy, contributing approximately 31–40% of global tea production and employing more than 1.1 million workers. India is among the leading tea-producing countries alongside China, Sri Lanka, Kenya, and Indonesia, with China being the largest producer [1, 2]. Assam and West Bengal are the primary tea-producing states, while Darjeeling and the Terai–Dooars region of Northern part of West Bengal state are internationally recognized for producing high-quality orthodox, green, and black tea varieties [3, 4]. Tea cultivation and processing generate extensive direct and indirect employment, particularly in rural and economically disadvantaged regions where plantations are concentrated [5, 6].

Tea production is a labour-intensive agro-based industry that relies heavily on manual activities such as cultivation, plucking, pruning, harvesting, and processing. Workers are frequently exposed to adverse climatic conditions, tea dust, pesticides, repetitive physical labour, and poor ergonomic practices [7, 8]. Tea garden workers frequently experience socio-economic disadvantage, limited occupational protection, and restricted access to healthcare services despite their important role in tea production [9]. Low wages, poor living conditions, food insecurity, limited social mobility, and inadequate occupational safety measures further increase their vulnerability [9, 10]. In addition, the remoteness of tea estates and poor healthcare infrastructure limit access to timely medical services, especially during emergencies [11].

The tea sector is also the largest organized employer of women workers in India, with women forming the backbone of plantation activities, especially tea plucking and maintenance work [11]. Previous studies have reported that women tea garden workers often experience lower educational attainment, limited economic opportunities, and greater occupational vulnerability compared with men [12, 13]. Men, on the other hand, are generally involved in comparatively heavier activities such as irrigation, spraying, and ploughing. The physically demanding nature of plantation work, combined with inadequate safety awareness and limited technical support, contributes to high levels of fatigue, musculoskeletal disorders, respiratory problems, and other occupational health concerns among tea garden workers [13–14].

Tea garden workers represent a vulnerable occupational population exposed to multiple health risks arising from hazardous working conditions, chemical exposures, poor sanitation, inadequate housing, food insecurity, and limited access to healthcare services [15–18]. Previous studies in tea plantation settings have documented a substantial burden of morbidity and poor access to healthcare among tea garden communities [19, 20]. Despite the economic importance of the tea industry in West Bengal, evidence regarding the combined assessment of morbidity patterns and healthcare utilization among tea garden workers in the Siliguri region remains limited. Most available studies have focused either on occupational health conditions or healthcare access separately, with limited examination of how socio-demographic characteristics, occupational exposures, and healthcare accessibility collectively influence worker health outcomes [21, 22]. Occupational health, as defined by the World Health Organization (WHO), refers to the promotion and maintenance of the highest degree of physical, mental, and social well-being among workers [11]. In tea plantations, workers’ health outcomes are influenced not only by occupational exposures but also by socio-economic determinants such as wages, nutrition, sanitation, housing, healthcare facilities, and awareness of welfare schemes [23, 24]. Tea garden workers in West Bengal remain particularly vulnerable to infectious diseases, chronic illnesses, malnutrition, and work-related injuries [25]. Limited access to healthcare, inadequate implementation of labour welfare policies, and low awareness of government health schemes further hinder healthcare utilization among this population [26].

Although the tea industry contributes significantly to the economy of West Bengal and India, research examining morbidity patterns and healthcare utilization among tea garden workers remains limited. Morbidity patterns serve as an important proxy for assessing a population’s health status and identifying disease burden and healthcare needs [27]. Understanding the prevalence of diseases, occupational health risks, and barriers to healthcare access is essential for designing effective interventions and improving the quality of life of this marginalized workforce. Therefore, this study aimed to assess morbidity patterns and healthcare utilization among tea garden workers in Siliguri, West Bengal, and to identify socio-demographic, occupational, and healthcare-access factors independently associated with morbidity burden and healthcare-seeking behaviour [28]. Nevertheless, the tea garden workers represent a vulnerable occupational population exposed to multiple health risks arising from occupational exposures, socioeconomic disadvantage, and barriers to healthcare access. Understanding these factors is important for informing evidence-based occupational health and public health interventions.

## Methods

### Study aim, design, and setting

This cross-sectional study aimed to assess morbidity patterns, explore healthcare utilization, and analyse factors influencing these aspects among tea garden workers in Siliguri, West Bengal. A quantitative cross-sectional research design was employed using a multistage random sampling approach. The study was conducted at the Taiphoo Tea Estate, a prominent tea garden in West Bengal that contributes significantly to the Terai tea industry. The tea gardens in this region are located in suburban and rural areas, often far from the main city, making healthcare access and utilization key concerns for workers. Siliguri, an urban centre with a population exceeding 7,00,000, has experienced rapid demographic expansion due to its strategic location and economic opportunities. The region is home to ethnically diverse populations, including Bengalis, Nepalis, Biharis, Marwaris, and indigenous tribal groups, many of whom are engaged in tea cultivation, plucking, and processing. These workers face occupational challenges, socio-economic disadvantages, and limited access to healthcare services.

### Study population and sampling

The study population comprised male and female tea garden workers aged 18–60 years who had been employed on tea plantations for at least one year prior to data collection. Individuals unwilling to participate were excluded from the study. A multistage random sampling approach was adopted. Initially, tea gardens within the Siliguri district were identified, and the study site was selected. Within the selected tea garden, eligible workers were identified from employment registers, and participants were selected using computer-generated random numbers.

Data were collected using a semi-structured questionnaire developed following an extensive review of the literature. The questionnaire included sections on socio-demographic characteristics, occupational exposures, morbidity patterns, healthcare utilization, health-seeking behaviour, and barriers to healthcare access. The questionnaire was reviewed by public health experts and pilot-tested prior to implementation. The sample size was estimated using the standard formula for prevalence studies, n = Z²p(1 − p)/d², assuming a prevalence of 50%, a 95% confidence level, and a precision of 8%. The minimum required sample size was estimated at 150 participants. After accounting for a 10% non-response rate, the final sample size was set at 167 participants.

### Ethical considerations

Ethical approval was obtained from the Institutional Ethics Committee (KMC IEC2 :696/2023) before data collection. Permissions were secured from the Doars Branch of the India Tea Association (DBITA), the tea estate secretary, and the labour welfare officer. Participation in the study was voluntary, and informed consent was obtained from all participants before interviews. The study’s purpose and procedures were explained in Bengali and Hindi, with information sheets and consent forms provided in both languages. All collected data were anonymized, and confidentiality was strictly maintained, with the research team serving as the sole custodian of the data.

### Data collection method

Following approval from the Institutional Ethics Committee, data collection was conducted between January and March 2024 at the selected tea estate in Siliguri, West Bengal. Eligible participants were identified from the tea garden worker registers and selected using computer-generated random numbers in accordance with the sampling strategy described above. Data were collected through face-to-face interviews using a pre-tested semi-structured questionnaire. Interviews were conducted by trained researchers who were familiar with the study objectives and data collection procedures. To ensure participant comprehension and accurate responses, interviews were conducted in the local languages commonly spoken by the workers, including Bengali and Hindi. Each interview lasted approximately 15–20 min. The questionnaire collected information on socio-demographic characteristics, occupational exposures, morbidity patterns, healthcare utilization, health-seeking behaviour, and barriers to accessing healthcare services. Responses were recorded electronically using a password-protected laptop to minimize data-entry errors and improve data management. Several measures were implemented to ensure data quality. Data collectors received training prior to fieldwork, and the questionnaire was pilot tested before the study commenced. Daily reviews of completed questionnaires were undertaken to identify incomplete or inconsistent responses. Any discrepancies were clarified immediately, where feasible, during the data collection period. To maintain confidentiality, no personally identifiable information was recorded in the analytical dataset. All electronic records were stored securely and were accessible only to the research team.

### Statistical analysis

Data analysis was performed using Jamovi version 2.3.28 and verified using Python-based statistical procedures. Descriptive statistics were used to summarize socio-demographic characteristics, morbidity prevalence, occupational exposures, and healthcare utilization patterns. Chi-square tests were used to examine associations between categorical variables. Fisher’s exact test was applied when expected cell frequencies were less than five, and the assumptions required for Chi-square testing were not satisfied. A cumulative morbidity burden score was calculated by summing the presence of reported morbidity conditions across infectious disease, chronic disease, musculoskeletal, respiratory, dermatological, disability, and occupational injury domains. Each reported condition contributed one point to the total score, with higher scores indicating greater overall morbidity burden. Multivariable regression analyses were conducted to identify factors independently associated with morbidity burden and healthcare utilization. A Poisson regression model with robust standard errors was used to examine predictors of cumulative morbidity burden, while binary logistic regression was used to identify predictors of healthcare utilization. Results are presented as adjusted incidence rate ratios (IRRs) and adjusted odds ratios (AORs) with corresponding 95% confidence intervals. All models were interpreted as adjusted associations because the cross-sectional design does not permit causal inference.

## Results

A total of 167 tea garden workers participated in the study. The largest proportion of workers belonged to the 41–50 years age group (37.7%), followed by those aged 31–40 years (29.9%) and 18–30 years (26.9%). The study population was predominantly female (71.9%). Educational attainment was generally low: 58.1% of participants were illiterate, and only 8.4% had completed high school. Most participants were employed as permanent workers (88.0%), although 62.9% reported never receiving formal safety training. The use of personal protective equipment was variable: gloves were the most commonly used protective measure (65.2%), whereas caps (15.2%) and face masks (19.7%) were used relatively infrequently. More than half of the workers (55.7%) had long-duration occupational exposure exceeding 20 years, indicating prolonged engagement in plantation-related work and potential cumulative exposure to occupational hazards (Table 1).

**Table 1:** Participant characteristics and occupational details

Characteristic	Category	*N*	%
Total Participants	-	167	100.0
Age Group (Years)	18–30	45	26.9
	31–40	50	29.9
	41–50	63	37.7
	51–60	9	5.4
Gender	Male	47	28.1
	Female	120	71.9
Education Level	Illiterate	97	58.1
	Primary Education	56	33.5
	High School	14	8.4
Employment Type	Permanent	147	88.0
	Temporary	12	7.2
	Contractual	8	4.8
Safety Training	Received	62	37.1
	Never Received	105	62.9
Protective Equipment Used	None	101	60.5
	Hand gloves	109	65.2
	Caps	25	15.2
	Face masks	33	19.7
Occupational Exposure Duration	Short-duration occupational exposure (1–10 years)	25	15.0
	Medium-duration occupational exposure (11–20 years)	49	29.3
	Long-duration occupational exposure (> 20 years)	93	55.7

The study revealed a substantial burden of infectious, chronic, occupational, and work-related health conditions among tea garden workers. Among infectious diseases, self-reported tuberculosis was the most frequently reported condition, affecting 50.9% of participants, followed by diarrhoea or water-related gastrointestinal illness (20.6%). Other reported infectious conditions included skin infections (11.1%), eye infections characterized by redness and discharge (10.8%), and worm infestations (6.6%). Among chronic conditions, self-reported diabetes mellitus was the most common (36.5%), followed by self-reported hypertension (16.2%). Participants also reported chest pain with respiratory symptoms (12.0%), chronic gastrointestinal complaints (9.6%), self-reported anaemia (8.2%), and chronic respiratory complaints (5.4%). Uterine disorders were reported by 3.6% of female participants. Work-related musculoskeletal disorders were highly prevalent. More than half of the workers (52.7%) reported muscle and joint pain or discomfort, while 32.3% experienced backache and 11.4% reported tingling or numbness in the hands and thumbs. These findings highlight the physically demanding nature of plantation work and the potential ergonomic challenges associated with repetitive occupational activities. Skin and respiratory conditions were also common. Long-lasting burning sensations and cracked skin were reported by 33.5% of workers, while 25.7% experienced frequent itchiness and redness.

Chemical irritations (22.8%) and allergic reactions to plants (18.0%) were also frequently reported. Respiratory symptoms were widespread, with 54.5% of workers reporting cough with mucus and 41.3% reporting breathing difficulties. Occupational injuries represented the most frequently reported category of work-related health problems. More than two-thirds of workers (67.7%) reported injuries related to slips, falls, lacerations, electrical incidents, heavy loads, or machinery-related accidents. In addition, 26.9% reported insect or animal bites, while 5.4% experienced lightning-related accidents. Overall, the findings demonstrate a high burden of infectious diseases, chronic conditions, musculoskeletal complaints, respiratory symptoms, skin disorders, and occupational injuries among tea garden workers, underscoring the need for strengthened occupational health measures, workplace safety interventions, and improved access to healthcare services (Table 2).

**Table 2:** Distribution of participants based on their morbidity patterns

Category	Morbidity Type	*N*	%
Infectious Diseases	Self-reported tuberculosis	85	50.9
	Diarrhoea or stomach problems from water	34	20.6
	Skin infections with easy spread	19	11.1
	Red eye, frequent eye discharge	18	10.8
	Worm infestations	11	6.6
Chronic Diseases	Self-reported diabetes mellitus	61	36.5
	Self-reported hypertension	27	16.2
	Chest pain with respiratory symptoms	20	12.0
	Chronic respiratory complaints	9	5.4
	Chronic gastrointestinal complaints	16	9.6
	Self-reported anaemia	14	8.2
	Fatigue, body aches, headache, stress	14	8.4
	Uterine disorders (female participants)	6	3.6
Musculoskeletal Disorders	Pain or discomfort in muscles or joints	88	52.7
	Tingling sensations in hands, numbness in the thumb	19	11.4
	Swollen or painful veins in legs	6	3.6
	Backache	54	32.3
Skin Disorders	Frequent itchiness and redness	43	25.7
	Allergic reactions to plants	30	18.0
	Irritations from chemicals	38	22.8
	Long-lasting burning sensations and cracked skin	56	33.5
Respiratory Disorders	Breathing difficulties	69	41.3
	Cough and mucus	91	54.5
	Lung infections	7	4.2
Injuries/Accidents	Occupational injuries (slips, falls, lacerations, electrical, heavy loads, machinery)	113	67.7
	Insect/animal bites	45	26.9
	Lightning accidents	9	5.4

Healthcare utilization patterns were assessed among workers who reported morbidity during the reference period. Of the 146 workers experiencing health-related symptoms, 70.5% sought care from a healthcare facility, while 29.5% did not seek formal medical attention. Tea garden hospitals represented the most frequently utilized healthcare facility (56.3%), followed by block hospitals (20.4%) and primary health centres (19.4%). District hospitals and private hospitals were infrequently utilized (1.9% each). Very few participants reported seeking care from district hospitals or private healthcare facilities, indicating a strong reliance on healthcare services available within or near the plantation setting. The most common reasons for seeking healthcare were musculoskeletal complaints (39.8%) and skin or allergic conditions (35.0%), followed by injuries and accidents (10.7%). Respiratory symptoms, headaches, and acute febrile illnesses accounted for a smaller proportion of healthcare visits. These findings are consistent with the occupational profile of tea plantation work, which involves repetitive physical activity, prolonged environmental exposure, and frequent contact with vegetation and agricultural chemicals.

Accessibility appeared to influence healthcare utilization. More than half of the participants (52.5%) reached healthcare facilities within 10–25 min, while approximately one-third required 25–40 min to travel. Public transportation was the most commonly used mode of travel (58.3%), suggesting continued reliance on shared transport systems to access healthcare services. Prescription medications constituted the most frequently reported form of treatment (55.3%), followed by referrals to tertiary healthcare facilities (28.2%). However, healthcare experiences were mixed, with almost equal proportions of workers reporting satisfaction (51.5%) and dissatisfaction (48.5%) with the services received. The relatively high level of dissatisfaction may indicate gaps in quality of care, treatment effectiveness, accessibility, or continuity of services. Medication adherence was suboptimal. Only 37.9% of participants reported taking prescribed medications regularly, whereas more than half (51.5%) reported irregular adherence, and 10.7% did not purchase prescribed medicines. These findings suggest the presence of barriers that may limit treatment compliance and long-term disease management. Utilization of government healthcare schemes was also low. Only 20.4% of participants reported benefiting from government-supported health programmes, whereas nearly four-fifths (79.6%) reported no utilization of such schemes. This pattern suggests limited awareness, accessibility, or uptake of available public health entitlements among tea garden workers.

Overall, the findings indicate that although most workers sought healthcare when ill, healthcare utilization remained heavily dependent on tea garden hospitals, medication adherence was inconsistent, and utilization of government health schemes was limited. These results highlight the need to strengthen healthcare accessibility, improve continuity of care, enhance awareness of government health programs, and address barriers to treatment adherence among tea garden workers (Table 3).

**Table 3:** Patterns of healthcare utilization and health-seeking behaviour among tea garden workers

Pattern of Healthcare utilization	Category	*N*	%
Visited a healthcare facility	Yes	103	70.5
	No	43	29.5
Facility type (*N* = 103)	Tea garden hospital	58	56.3
	Block hospital	21	20.4
	Primary health centre	20	19.4
	District hospital	2	1.9
	Private hospital	2	1.9
Reasons for visit	Musculoskeletal issues	41	39.8
	Skin/allergic reactions	36	35.0
	Injuries/accidents	11	10.7
	Respiratory issues	5	4.9
	Headaches	6	5.8
	Fever, cough, cold	4	3.9
Time taken to travel	10–25 min	53	52.5
	25–40 min	31	30.7
	40 min–1 h	12	11.9
	Up to 2 h	5	5.0
Mode of travel	Public transport	60	58.3
	Private vehicle	33	32.0
	Walked	10	9.7
Treatment received	Prescription medicines	57	55.3
	Referral to tertiary care	29	28.2
	Pathological tests	9	8.7
	First aid	8	7.8
Satisfaction with care	Satisfied	53	51.5
	Dissatisfied	50	48.5
Medication adherence	Regularly	39	37.9
	Not regularly	53	51.5
	Did not purchase medication	11	10.7
Utilization of government schemes	Yes	21	20.4
	No	82	79.6

### Predictors of cumulative morbidity burden

An adjusted Poisson regression model was used to identify factors independently associated with cumulative morbidity burden among tea garden workers (Table 4). After adjustment for socio-demographic, occupational, and healthcare-related factors, female workers had a significantly higher morbidity burden than male workers (IRR = 1.35, 95% CI: 1.14–1.60, *p* < 0.001). Occupational chemical exposures emerged as the strongest predictors of morbidity burden. Workers exposed to herbicides had significantly greater morbidity burden than non-exposed workers (IRR = 2.53, 95% CI: 2.08–3.08, *p* < 0.001). Similarly, exposure to chemical fertilizers was associated with substantially higher morbidity burden (IRR = 2.77, 95% CI: 2.12–3.62, *p* < 0.001). These findings suggest that prolonged occupational exposure to agricultural chemicals may contribute to the accumulation of multiple health conditions among tea garden workers. The use of personal protective equipment was also significantly associated with morbidity burden (IRR = 1.28, 95% CI: 1.07–1.53, *p* = 0.007). This finding should be interpreted cautiously because workers with greater occupational exposure or existing health problems may be more likely to use protective equipment. Better access to primary healthcare facilities was positively associated with the morbidity burden (IRR = 1.22, 95% CI: 0.99–1.51, *p* = 0.067). In contrast, age, educational status, monthly income, and receipt of safety training were not independently associated with cumulative morbidity burden after adjustment. Overall, the findings indicate that occupational chemical exposure represents a key factor associated with morbidity burden among tea garden workers.

**Table 4:** Adjusted Poisson regression analysis of factors associated with cumulative morbidity burden among tea garden workers

Predictor	Adjusted IRR	95% CI	*p*-value
Female sex	1.35	1.14–1.60	< 0.001
Age > 40 years	1.06	0.93–1.22	0.364
Illiterate	1.07	0.95–1.22	0.260
Low monthly income ( < = Rs 8,600)	1.02	0.89–1.16	0.799
Herbicide exposure	2.53	2.08–3.08	< 0.001
Chemical fertilizer exposure	2.77	2.12–3.62	< 0.001
Received safety training	0.86	0.71–1.05	0.144
Use of any PPE	1.28	1.07–1.53	0.007
Better PHC access (rating > = 4)	1.22	0.99–1.51	0.067

Estimates are adjusted for age and gender

*IRR *Incidence Rate Ratio, *CI* Confidence Interval, *PHC* Primary Health Centre, *PPE* Personal Protective Equipment

Statistical significance was defined as *p* < 0.05

Among workers who reported morbidity or symptoms during the previous six months (*n* = 139), widowed workers had significantly higher odds of visiting a healthcare facility compared with married workers (AOR = 5.07, 95% CI: 1.40–18.31, *p* = 0.013). Similarly, workers with low monthly income (≤ ₹8,600) were about five times more likely to utilize healthcare services than those with higher income levels (AOR = 4.99, 95% CI: 1.99–12.49, *p* < 0.001). Female workers had lower odds of healthcare utilization than male workers (AOR = 0.44, 95% CI: 0.17–1.16), although this association did not reach statistical significance (*p* = 0.097). Likewise, illiterate workers showed lower odds of seeking healthcare compared with literate workers (AOR = 0.46, 95% CI: 0.20–1.08, *p* = 0.075), but the association was not statistically significant. Age above 40 years, receipt of safety training, use of personal protective equipment (PPE), and morbidity burden score were not significantly associated with healthcare utilization (all *p* > 0.05). Although PPE users appeared less likely to visit healthcare facilities (AOR = 0.49), the confidence interval included the null value, indicating insufficient evidence for a significant association. Overall, after adjusting for potential confounders, widowed marital status and low monthly income emerged as independent predictors of healthcare utilization among symptomatic workers (Table 5).

**Table 5:** Adjusted logistic regression analysis of factors associated with healthcare utilization among symptomatic tea garden workers

Predictor	Adjusted OR	95% CI	*p*-value
Female sex	0.44	0.17–1.16	0.097
Age > 40 years	0.76	0.32–1.80	0.532
Widowed	5.07	1.40-18.31	0.013
Illiterate	0.46	0.20–1.08	0.075
Low monthly income ( < = Rs 8,600)	4.99	1.99–12.49	< 0.001
Received safety training	0.88	0.26–3.02	0.843
Use of any PPE	0.49	0.17–1.40	0.181
Morbidity burden score	0.91	0.79–1.05	0.205

Outcome = healthcare facility visit among workers reporting morbidity/symptoms in the previous six months (*N* = 139)

*AOR* adjusted odds ratio, *CI *confidence interval, *PPE *personal protective equipment

Statistical significance at *p* < 0.05

Table 6 presents the association between selected morbidity symptoms and healthcare utilization among workers using Fisher’s exact test. Workers reporting musculoskeletal symptoms were significantly more likely to seek healthcare compared with those who did not report such symptoms (69.9% vs. 0.0%; OR = 30.01, 95% CI: 1.65–545.46, *p* = 0.001). Similarly, workers with respiratory symptoms had significantly higher odds of healthcare utilization than those without respiratory symptoms (71.3% vs. 10.0%; OR = 22.38, 95% CI: 2.74–182.92, *p* < 0.001). No statistically significant associations were observed between healthcare utilization and skin disorders (OR = 1.47, 95% CI: 0.71–3.02, *p* = 0.353), occupational injury (OR = 0.82, 95% CI: 0.33–2.05, *p* = 0.821), tuberculosis (OR = 1.07, 95% CI: 0.52–2.17, *p* = 1.000), or diabetes (OR = 1.07, 95% CI: 0.53–2.16, *p* = 1.000). These findings suggest that workers experiencing musculoskeletal and respiratory symptoms were more likely to seek healthcare services, whereas healthcare utilization did not differ significantly according to the presence of skin disorders, occupational injuries, tuberculosis, or diabetes.

**Table 6:** Fisher’s exact analysis of morbidity symptoms and healthcare utilization

Morbidity/symptom	Sought care among exposed *n*/*N* (%)	Sought care among unexposed *n*/*N* (%)	Crude OR	95% CI	Fisher exact *p*-value
Musculoskeletal symptoms	93/133 (69.9)	0/6 (0.0)	30.01	1.65-545.46	0.001
Respiratory symptoms	92/129 (71.3)	1/10 (10.0)	22.38	2.74-182.92	< 0.001
Skin disorders	61/87 (70.1)	32/52 (61.5)	1.47	0.71–3.02	0.353
Any occupational injury	74/112 (66.1)	19/27 (70.4)	0.82	0.33–2.05	0.821
Tuberculosis	54/80 (67.5)	39/59 (66.1)	1.07	0.52–2.17	1.000
Diabetes	48/71 (67.6)	45/68 (66.2)	1.07	0.53–2.16	1.000

Fisher’s exact test was used because of small cell counts. Exposed = presence of the specified morbidity/symptom; unexposed = absence of the specified morbidity/symptom

*OR *odds ratio, *CI *confidence interval

Statistical significance at *p* < 0.05

## Discussion

The present study highlights the substantial burden of morbidity and occupational health challenges among tea garden workers. The findings demonstrate that infectious diseases, chronic illnesses, musculoskeletal disorders, respiratory symptoms, skin conditions, and occupational injuries remain common among workers, reflecting the persistent health vulnerabilities experienced by plantation communities. These findings are consistent with previous studies conducted in tea-growing regions of India, Bangladesh, Nepal, and Sri Lanka, which have documented poor health outcomes among tea plantation workers due to hazardous working conditions, poverty, inadequate occupational protection, and limited access to healthcare [3, 5, 20, 22]. Women constituted the majority of the workforce and experienced significantly greater cumulative morbidity burden than men. This finding is consistent with previous studies among tea plantation communities that have highlighted the multiple occupational and social disadvantages experienced by women workers, including lower educational attainment, nutritional vulnerabilities, and greater domestic responsibilities in addition to plantation work [13, 23]. The higher morbidity burden observed among women may be related to prolonged occupational exposure, repetitive manual tasks, nutritional vulnerabilities, and the dual responsibilities associated with paid work and domestic caregiving. Given that women represent the backbone of tea plantation labour, their health status has important implications for both household welfare and plantation productivity.

A striking finding of the present study was the high prevalence of musculoskeletal complaints. More than half of the workers reported muscle and joint pain, while nearly one-third experienced backache. These findings align with previous evidence demonstrating a high prevalence of work-related musculoskeletal disorders among tea plantation workers and agricultural labourers exposed to repetitive movements, prolonged standing, awkward postures, and manual load carrying [8, 26, 29]. Tea plucking requires repetitive upper-limb activity and prolonged static postures, which may contribute to cumulative strain on the musculoskeletal system. The fact that musculoskeletal symptoms were also among the most common reasons for healthcare visits suggests that these conditions substantially affect daily functioning and work performance. Respiratory symptoms were also highly prevalent, with more than half of the participants reporting cough with mucus and over two-fifths reporting breathing difficulties. Similar findings have been reported among plantation workers exposed to agricultural dust, pollen, and chemical agents [28, 30]. Respiratory symptoms may reflect chronic exposure to environmental irritants and agrochemicals commonly encountered during plantation activities. Importantly, workers reporting respiratory symptoms were significantly more likely to seek healthcare services, suggesting that respiratory illnesses may be perceived as serious conditions that directly interfere with work capacity.

The study also revealed a considerable burden of dermatological conditions, including itching, redness, chemical irritation, and allergic reactions. Previous studies among tea plantation workers in Nepal and other tea-growing regions have documented similar patterns of skin disorders resulting from frequent contact with tea plants, fertilizers, pesticides, and environmental allergens [12, 16]. Although skin conditions were common, they were not significantly associated with healthcare utilization in the present study. This finding may indicate normalization of minor skin complaints among workers or the use of self-management strategies rather than formal healthcare seeking. Occupational injuries emerged as one of the most frequently reported work-related health problems. More than two-thirds of workers reported injuries associated with slips, falls, machinery, heavy loads, or other workplace hazards. Similar injury patterns have been documented among agricultural workers in South Asia, where inadequate workplace safety measures and physically demanding work environments contribute to high injury rates [24, 30]. The persistence of such injuries highlights the continuing need for occupational safety interventions within plantation settings.

One of the most important findings of this study was the strong association between occupational chemical exposures and the cumulative burden of morbidity. Workers exposed to herbicides and chemical fertilizers demonstrated substantially higher morbidity burden than unexposed workers. This observation is consistent with previous research linking long-term agrochemical exposure with adverse respiratory, dermatological, neurological, and systemic health outcomes among agricultural and plantation workers [28, 30]. The findings reinforce concerns regarding the health consequences of repeated chemical exposure and emphasize the importance of safer handling practices, effective risk communication, and occupational health surveillance. Interestingly, workers who reported using personal protective equipment had a higher morbidity burden. Similar observations have occasionally been reported in occupational studies, in which workers with greater exposure to hazards or pre-existing health problems are more likely to adopt protective measures [24, 28]. Therefore, this finding should not be interpreted as evidence that PPE increases disease risk. Rather, it may reflect reverse causality, whereby workers experiencing health problems become more likely to use protective equipment or are assigned to higher-risk tasks requiring PPE.

The healthcare utilization findings provide valuable insight into health-seeking behaviour within tea garden communities. Approximately 70% of symptomatic workers sought healthcare services, with tea garden hospitals serving as the primary source of care. This pattern is consistent with previous studies indicating that plantation-based healthcare facilities often represent the first point of contact for tea garden workers because of their proximity, affordability, and familiarity [2, 10]. However, despite relatively high healthcare utilization, substantial gaps remain. Nearly half of healthcare users reported dissatisfaction with services, medication adherence was poor, and utilization of government health schemes was remarkably low. Similar barriers to effective healthcare utilization have been documented among tea plantation populations, where socioeconomic disadvantage, limited awareness, and inadequate healthcare infrastructure often restrict access to comprehensive care [10, 23].

The finding that low-income workers were significantly more likely to seek healthcare may initially appear counterintuitive. However, this association may reflect a greater burden of illness among economically disadvantaged workers, leading to increased healthcare needs. Previous studies have consistently demonstrated strong links between poverty, undernutrition, morbidity, and healthcare utilization among marginalized populations [20, 31]. Similarly, widowed workers demonstrated significantly greater healthcare utilization, which may reflect increased health needs, greater perceived vulnerability, or reduced availability of family support during illness. Finally, the study identified musculoskeletal and respiratory symptoms as the strongest morbidity-related drivers of healthcare utilization. These conditions directly affect physical functioning and work productivity and may therefore prompt workers to seek treatment more readily than chronic illnesses or less disabling conditions. In contrast, healthcare utilization did not differ significantly according to the presence of diabetes, tuberculosis, skin disorders, or occupational injuries. This finding raises concerns regarding delayed care-seeking for chronic and potentially serious conditions and suggests the need for improved health awareness and routine screening programmes within plantation communities. Overall, the findings indicate that tea garden workers continue to experience a substantial burden of occupational and non-occupational morbidity. While healthcare services are available and utilized by many workers, significant challenges remain in reducing occupational exposures, strengthening preventive health measures, improving quality of care, and ensuring equitable access to healthcare resources. Addressing these challenges will require coordinated action involving plantation management, public health authorities, healthcare providers, and labour welfare agencies.

An important contribution of the present study is the identification of factors independently associated with morbidity burden and healthcare utilization among tea garden workers. The adjusted analyses demonstrated that occupational chemical exposures were strongly associated with cumulative morbidity burden, while socioeconomic factors such as income and marital status influenced healthcare utilization. These findings support a social determinant of health perspective, suggesting that worker health is shaped not only by individual characteristics but also by workplace exposures and access to healthcare resources. Nevertheless, these findings highlight the importance of integrating occupational health surveillance into routine primary healthcare services available within tea plantation settings. Such integration may facilitate earlier identification of work-related illnesses and strengthen preventive interventions among vulnerable worker populations.

### Practical implications

The findings of this study have several practical implications for occupational health policy and healthcare delivery in tea plantation settings. First, the strong association between herbicide and fertilizer exposure and cumulative morbidity burden highlights the urgent need to strengthen occupational safety practices within tea gardens. Regular risk assessments, safer chemical handling procedures, adequate provision of personal protective equipment, and periodic safety training should be prioritized to reduce hazardous exposures and prevent long-term health consequences among workers. Second, the high prevalence of musculoskeletal disorders, respiratory symptoms, and skin conditions suggests that routine occupational health screening should be integrated into existing plantation healthcare services. Early detection and timely management of these conditions could reduce disease progression, improve quality of life, and minimize productivity losses associated with work-related illness. Ergonomic interventions, including task rotation, improved work practices, and worker education on safe body mechanics, may further reduce the burden of musculoskeletal disorders.

Third, although a majority of symptomatic workers sought healthcare, the substantial levels of dissatisfaction with healthcare services, poor medication adherence, and low utilization of government health schemes indicate important gaps in healthcare delivery. Strengthening linkages between tea garden hospitals and the public healthcare system may improve referral mechanisms, continuity of care, and access to specialist services. Efforts to improve awareness and uptake of government-sponsored health programmes may also enhance financial protection and healthcare accessibility among workers and their families.

The finding that women experienced significantly higher morbidity burden underscores the importance of adopting gender-responsive occupational health strategies. Health programmes targeting tea garden workers should incorporate regular screening, health education, and preventive services that address the specific needs of women, who constitute the majority of the plantation workforce. Finally, the study highlights the need for a multisectoral approach involving plantation management, public health authorities, labour welfare departments, and healthcare providers. Improving worker health requires not only access to healthcare services but also safer working conditions, stronger occupational health regulations, effective health promotion activities, and social support mechanisms. Such interventions have the potential to improve worker well-being, enhance productivity, and help reduce health inequities within tea plantation communities.

### Strengths and limitations

This study provides a comprehensive assessment of morbidity patterns, occupational risk factors, and healthcare utilization among tea garden workers, a population that remains underrepresented in occupational health research despite its substantial contribution to the tea industry. By examining both health outcomes and healthcare-seeking behaviour, the study offers a broader understanding of the complex interplay between occupational exposures, disease burden, and access to care within plantation communities. The use of multivariable regression models further enabled the identification of independent predictors of cumulative morbidity burden and healthcare utilization.

However, several limitations should be considered when interpreting the findings. First, the cross-sectional design precludes establishing temporal or causal relationships between occupational exposures and health outcomes. Consequently, it cannot be determined whether the identified risk factors directly caused the observed morbidities. Second, several health conditions and occupational exposures were self-reported and may therefore be subject to recall bias, reporting bias, or misclassification. Although self-reported information remains valuable in community-based occupational health research, objective clinical assessments and environmental exposure measurements would have strengthened the findings. Third, the study was conducted among workers from selected tea gardens, which may limit the generalizability of the results to other plantation settings with different socio-economic, environmental, or healthcare contexts. Fourth, information on the intensity and duration of specific occupational exposures, including chemical exposure levels, was not available. Finally, unmeasured factors such as nutritional status, psychosocial stressors, workplace organization, and health literacy may have influenced both morbidity burden and healthcare utilization and could not be fully accounted for in the analysis. Also, several morbidity indicators, including tuberculosis, diabetes, hypertension, and other chronic conditions, were self-reported and were not independently verified through clinical records or laboratory investigations.

Despite these limitations, the study provides important evidence regarding the health challenges faced by tea garden workers and contributes to the limited literature on occupational health and healthcare utilization in plantation communities.

## Conclusion

Tea garden workers in West Bengal experience a substantial burden of infectious diseases, chronic illnesses, musculoskeletal disorders, respiratory symptoms, skin conditions, and occupational injuries. Occupational exposure to herbicides and chemical fertilizers emerged as important determinants of cumulative morbidity burden, while female workers experienced significantly greater overall morbidity. Although a majority of symptomatic workers sought healthcare, utilization remained heavily dependent on tea garden hospitals and was accompanied by low uptake of government health schemes, poor medication adherence, and considerable dissatisfaction with healthcare services.

The findings highlight the continuing influence of occupational and social determinants on worker health and underscore the need for integrated approaches that combine workplace safety measures, preventive healthcare services, and improved healthcare accessibility. Strengthening occupational health programmes, reducing hazardous exposures, improving treatment continuity, and enhancing awareness of available healthcare resources may contribute to better health outcomes among tea garden workers. Coordinated action involving plantation management, public health authorities, healthcare providers, and labour welfare agencies will be essential for addressing the health inequities experienced by this vulnerable workforce.

## Supplementary Information

Supplementary Material 1.

## Data Availability

The datasets generated and/or analyzed during the current study are not publicly available due to confidentiality and institutional restrictions.

## Abbreviations

AOR: Adjusted odds ratio
BMC: BioMed Central
CI: Confidence interval
IEC: Institutional Ethics Committee
IRR: Incidence rate ratio
OR: Odds ratio
PHC: Primary Health Centre
PPE: Personal Protective Equipment
TB: Tuberculosis
WHO: World Health Organization
