# Supplementary material for: A study on the morbidity patterns and healthcare utilization of tea garden workers of Siliguri, West Bengal, India
Source: BMC Public Health. 2026 Jul 31;26:3218. doi: 10.1186/s12889-026-28744-9 (PMC13737900; doi:10.1186/s12889-026-28744-9)
Supplement: Supplementary file 1 [file 12889_2026_28744_MOESM1_ESM.docx]

# **Title**: A A study on the morbidity patterns and healthcare utilization of tea garden workers of Siliguri, North Bengal, India

**QUESTIONNAIRE**

**Domain 1: Sociodemographic details**

1.1. ID No-

1.2 Gender: (a) Female (b) Male (c) Others

1.3. Age-

1.4. Marital status: (a) Unmarried (b) Married (c) Widowed (d) Others

1.5. Number of family members living with you in this tea garden-

1.6. Number of family members employed in this tea garden-

1.7. Number of family members employed in any other sector-

1.8. Monthly income of your family-

1.9. Educational level: (a) Illiterate (b) Primary level (c) High school level (d) Others, please specify

1.10. Tobacco consumption: (a) Smoking (b) Smokeless (c) Both (d) None

1.11. Alcohol consumption: (a) Regularly (b) Occasionally (c) Never

**Domain 2: Occupational details**

2.1. Nature of employment: (a) Permanent (b) Contractual (c) Others (Specify if any)

2.2. Years of employment in the tea sector-

2.3. Work hours per day-

2.4. Number of workdays per week-

2.5. Do you have to work in shifts? (a) Yes (b) No

2.6. Breaks: Duration:

2.7. Activities carried out per day: (a) Plucking (b) Weeding & Pruning (c) Planting and Transplanting (d) Carrying and Transporting (e) Others (Please specify if any)

2.8. Exposure to specific chemicals or materials used in tea garden agriculture-

| Exposure | Yes | No |
| --- | --- | --- |
| 1. Pesticides |  |  |
| 2. Herbicides |  |  |
| 3. Insecticides |  |  |
| 4. Tea processing chemicals |  |  |
| 5. Chemical fertilizers |  |  |
| 6. Others (Please specify if any) |  |  |

2.9. Did you receive any safety training related to your work? (a) Yes (b) No

2.10. Do you use any protective equipment? (a) Yes (b) No

- If the answer is Yes, what kind of equipment do you use while working? (You can select more than one option)

(a) Hand Gloves (b) Cap/Hat (c) Face Mask (d) Goggles (e) Chapel (f) Shoes (g) Others (Please specify if any)

**Domain 3: Self-reported morbidity**

3.1. Are you suffering from any health ailments?

| **Health ailment** | **Yes** | **No** |
| --- | --- | --- |
| 1. Infectious disease |  |  |
| 1.a. Malaria |  |  |
| 1. b. Dengue |  |  |
| 1. c. Tuberculosis |  |  |
| 1.d. Water-borne diseases |  |  |
| 1.e. Others (please specify) |  |  |
| 2. Chronic disease |  |  |
| 2.a. Diabetes |  |  |
| 2. b. Hypertension |  |  |
| 2. c. Heart disease |  |  |
| 2.d. Chronic lung disease |  |  |
| 2.e. Cancer |  |  |
| 2. f. Others (Please specify) |  |  |
| 3. No health ailments |  |  |

3.2. Do you have any known disability?

| **Disability** | **Yes** | **No** |
| --- | --- | --- |
| 1. Visual |  |  |
| 2. Locomotor |  |  |
| 3. Hearing |  |  |
| 4. Memory |  |  |
| 5. Loss of limb |  |  |
| 6. Others (Please specify) |  |  |

3.3. Have you had any health symptoms/conditions over the last 6 months?

**1. Musculoskeletal Disorders**

| **Symptoms/Conditions** | **Yes** | **No** |
| --- | --- | --- |
| 1. Pain/discomfort in neck, shoulder, hand, back, etc.) |  |  |
| 2. Carpel Tunnel Syndrome (tingling, weakness in thumb) |  |  |
| 3. Varicose veins |  |  |
| 4. Others (Please specify) |  |  |

**2. Skin problems/allergic reactions**

| **Symptoms/Conditions** | **Yes** | **No** |
| --- | --- | --- |
| 1. Contact dermatitis  (Redness, blisters, burning sensation, etc. on the skin) |  |  |
| 2. Allergic reaction to plants |  |  |
| 3. Chemical irritations |  |  |
| 4. Others (Please specify) |  |  |

**3. Respiratory problems**

| **Symptoms/Conditions** | **Yes** | **No** |
| --- | --- | --- |
| 1. Difficulty breathing |  |  |
| 2. Coughing, phlegm |  |  |
| 3. Others (Please specify) |  |  |

**4. Injuries/Accidents**

| **Symptoms/Conditions** | **Yes** | **No** |
| --- | --- | --- |
| 1. Slips or Falls |  |  |
| 2. Lacerations/cuts |  |  |
| 3. Insect/animal bites |  |  |
| 4. Others (Please specify) |  |  |

**Domain 4: Healthcare utilization**

***Part 1: For those who have suffered morbidity over the last 6 months***

4.1. Did you visit any healthcare facilities in the last 6 months?

(a) Yes (b) No

- If the answer is (a) what facility did you visit?

(a) Tea Garden hospital

(b) Sub-center

(c) Primary Health Center

(d) Community Health Center

(e) District Hospital

(f) Medical college and hospital

(g) Pharmacy/Drug store

(h) AYUSH Hospital

(i) Private hospital

(j) Private clinic

(k) Home visit

(l) Others

- If the answer is (b), mention reasons for not seeking healthcare during illness-

(a) The health facility is too far from my home (b) No transport facilities (c) I chose self-medication/home remedies (d) No paid leave (e) I went to a spiritual healer (e) I had no one to accompany me (f) I didn't think the illness was so serious (g)

4.2. What symptom/condition led you to visit the facility? (a) Musculoskeletal (Please specify) (b) Respiratory (Please specify) (c) Skin/Allergic reactions (Please specify) (d) Injuries/Accidents (Please specify)

4.3. What was the travel time to the facility?

4.4. How did you get there? (a) Private vehicle (b) Public transportation (c) Walked (d) Ambulance

4.5. What services did you receive at the facility during your last visit? (You can mention more than one)

4.6. Were you satisfied with the services you received? (a) Yes (b) No

- If the answer is No, mention the reasons-

(a) The doctor didn't check well (b) Unavailability of drugs (c) Delayed recovery (d) Services were very expensive (e) Others (Please specify if any)

4.7. Were you called for any follow-up visits? (a) Yes, was called (b) Was called and I went (c) Was called, but I did not go (d) No, was not called.

- If the answer is (c), mention reasons-

(a) Financial constraints (b) Transportation issues (c) Work-related obligations (d) Lack of prioritization (e) Poor treatment satisfaction (f) Others (Please specify if any)

4.8. Did you take your medication regularly according to the prescribed treatment? (a) Yes, regularly (b) Not regularly (c) Never (d) Did not purchase medication

4.9. Did you have any out-of-pocket expenses for public healthcare services? (a) Yes (b) No

4.10. Did you utilize any of the schemes provided by the government? (a) Yes (b) No

- If the answer is No, why did you not utilize any of the available health schemes?

(a) I was not aware (b) Was not informed at the facility (c) Personal belief (e) Others (Please specify if any)

***Part 2: For those who have not suffered morbidity over the last 6 months***

4.11. Do you seek healthcare when you have a health problem? (a) Always (b) Sometimes (c) Never

- If the answer is Never, mention the reasons-

(a) Health facility/healthcare provider is difficult to reach (b) No transport facilities (c) No paid leave (d) Seek home remedies/self-medication (e) Visit spiritual healers (f) No one to accompany

4.12. What facility do you visit?

| Facility | Yes | No |
| --- | --- | --- |
| 1. Tea Garden Hospital |  |  |
| 2. Sub-center |  |  |
| 3. Primary Health Center |  |  |
| 4. Community Health Center |  |  |
| 5. District hospital |  |  |
| 6. Medical college and hospital |  |  |
| 7. Pharmacy/Drug store |  |  |
| 8. AYUSH Hospital |  |  |
| 9. Private hospital |  |  |
| 10. Private clinic |  |  |
| 11. Home visit |  |  |
| 12. Others |  |  |

4.13. How often do you go for follow-up visits? (a) Always (b) Skip sometimes (c) Do not go

4.14. Do you take medicines as prescribed by the healthcare provider after your check-up?

(a) Always (b) Skip sometimes (c) Never

4.15. How often do you pay out-of-pocket for public healthcare services? (a) Always (b) Never, I use Government schemes

4.16. On what occasion do you seek healthcare?

(a) Health camps (b) Minor health issue (b) Vaccination drives (c) Severe or urgent health issue (d) When home remedies fail to work (e) Others (Please specify if any)

**Domain 5: Miscellaneous**

***Health Insurance***

5.1. What type of health insurance scheme do you utilize?

(a) Government Health Insurance Scheme (b) Private Health Insurance Scheme (c) Community-based Health Insurance Scheme (d) Employees State Insurance Scheme (ESIS) (d) Others (Please specify if any) (e) I have no health insurance

5.2. Have you ever utilized your health insurance? (a) Yes (b) No

***Food security***

5.3. How would you describe the availability of food in your household throughout the year?

(a) Always enough (b) Usually enough (c) Sometimes not enough (d) Often not enough (e) Never enough

5.4. Are there specific times of the year when it is more challenging for your household to access enough food? (a) Yes (b) No

- If the answer is Yes, mention the time of the year.

5.5. How would you describe the variety of foods available to your household regularly? (a) Varied (b) Somewhat varied (c) Limited Variety

5.6. Specify barriers to accessing food: (a) High food prices (b) No free rations (c) Limited access to markets (d) Transportation issues (e) Limited availability of food items.

5.7. Are there any organizations or community groups that provide food or nutritional support to you and your family? (a) Yes (b) No

5.8. Have you or your family benefited from such support? (a) Yes (b) No

***Social security***

5.9. On a scale of 1-5 please rate the quality and accessibility of the following provisions. (1 being very poor and 5 being excellent)

| **Provision** | **Rating** |
| --- | --- |
| 1. Housing |  |
| 2. Water resources |  |
| 3. Electricity |  |
| 4. Toilet facility |  |
| 5. Sanitation facility |  |
| 6. Primary health facilities |  |
| 7. Educational facilities |  |
| 8. Organizational support |  |
| 9. Recreational facility |  |

5.10. Are you aware of any financial assistance programs or microcredit initiatives available to tea garden workers for economic empowerment? (a) Yes (b) No

- If the answer is Yes, have you or your family members benefitted from such initiatives? (a) Yes (b) No

5.11. Do you have access to a banking or savings facility within the tea garden or nearby?

(a) Yes (b) No
